# Preventive Effect of Low Intensity Pulsed Ultrasound against Experimental Cerebral Ischemia/Reperfusion Injury via Apoptosis Reduction and Brain-derived Neurotrophic Factor Induction

**DOI:** 10.1038/s41598-018-23929-8

**Published:** 2018-04-03

**Authors:** Chang-Mu Chen, Cheng-Tien Wu, Ting-Hua Yang, Shing-Hwa Liu, Feng-Yi Yang

**Affiliations:** 1 Division of Neurosurgery, Department of Surgery, National Taiwan University Hospital and National Taiwan University, College of Medicine, Taipei, Taiwan; 2 Institute of Toxicology, College of Medicine, National Taiwan University, Taipei, Taiwan; 3 Department of Otolaryngology, National Taiwan University Hospital, Taipei, Taiwan; 4 Department of Pediatrics, College of Medicine and Hospital, National Taiwan University, Taipei, Taiwan; 5 Department of Medical Research, China Medical University Hospital, China Medical University, Taichung, Taiwan; 6 Department of Biomedical Imaging and Radiological Sciences, National Yang-Ming University, Taipei, Taiwan; 7 Biophotonics and Molecular Imaging Research Center, National Yang-Ming University, Taipei, Taiwan

## Abstract

Stroke is known as the top 10 causes of death worldwide. Development of effectively neuroprotective or preventive strategies for ischemia stroke is imperative. For the purpose of stroke prevention, we tested the neuroprotective effects of low-intensity pulsed ultrasound (LIPUS) on ischemic stroke. Adult C57BL/6 mice were used to daily treatment with LIPUS for 5 days on left hemisphere before middle cerebral artery occlusion (MCAO)-induced cerebral ischemia/reperfusion injury. Western blotting and immunohistochemistry were performed to assess the protein expressions of signaling molecules. Pretreatment with LIPUS significantly ameliorated the brain ischemic damage, including the reduction of neurological deficit score, infarct area, histopathological score, and showed a better performance in neurological and behavior functions. LIPUS pretreatment could also significantly decrease the neuronal cell apoptosis and upregulation of apoptosis-related signaling molecules and downregulation of brain-derived neurotrophic factor (BDNF) in brain tissues of MCAO-treated mice. Furthermore, LIPUS significantly prevented the decreased cell viability, the increased caspase-3 cleavage, and the decreased BDNF expression in ischemia/reperfusion-treated microglial cells. These results demonstrate that LIPUS effectively prevented the cerebral ischemia/reperfusion injury through apoptosis reduction and BDNF induction in a MCAO mouse model. The neuroprotective potential of LIPUS may provide a novel preventive strategy for ischemic stroke in high-risk patients.

## Introduction

Stroke is the fifth leading cause of death in the United States^1^ and the top 10 causes of death worldwide. The death globally caused by stroke is approximately 6.24 million in 2015^2^. and those who survived from stroke often remained some serious disability. Two main stroke types are known as hemorrhagic stroke and ischemic stroke; most patients are belong to ischemic stroke (87%)^3^. Currently, rarely pharmacological treatments can effectively ameliorate the sequelae from ischemic stroke. A promising and underway strategy to utilize thrombolytic therapies is tissue plasminogen activator (tPA) treatment^4^. However, the therapeutic time window of tPA has a restriction within 6 h. As a result, once happened, most stroke patients do not receive any specific pharmacology therapy, and only rehabilitative modalities would be expected to improve the functional outcomes. Therefore, to develop the potential neuroprotective or therapeutic strategies to effectively improve the sequelae during stroke is imperative.

Therapeutic ultrasound has been to obtain clinical acceptance. Ultrasound can transmit into a target tissue and produce physiological change through thermal or non-thermal effects^5^. Low-intensity pulsed ultrasound (LIPUS) belongs to an ultrasound with non-thermal mechanism and can output in a pulse wave mode that is delivered at a much lower intensity (<3 W/cm^2^) than traditional ultrasound energy^6^. LIPUS exerts the biological consequences through physical effects and training procedures. LIPUS is known to accelerate bone and tissue regeneration following injury^7,8^. In the brain, LIPUS has been indicated that presents positive effects on axonal regeneration in damaged nerves^9,10^. Transcranial pulsed ultrasound is capable of stimulating intact brain circuitry and promoting levels of brain-derived neurotrophic factor (BDNF)^11^. LIPUS treatment could also increase the protein levels of neurotrophic factors, which activate integrin receptor signaling to protect brain astrocytes against injury in an aluminum-induced brain disorder rat model^12^ and improve damages in the hippocampus and corpus callosum in a rat vascular dementia model^13^. These findings suggested that LIPUS potentially stimulated the release of neurotrophic factors to contract brain damages.

The cumulative evidence from animal and clinical studies indicated that ultrasound at lower energy levels (<2 W/cm^2^) enhanced enzymatic mediated thrombolysis in the treatment of ischemic stroke^14–16^. Moreover, the non-neuronal cells like as microglial cells and endothelial cells have been shown to produce substantial level of BDNF after ischemic stroke in rats^17^. Nevertheless, prevention is better than cure. In this study, therefore, we hypothesized that LIPUS pre-treatment possesses the preventive effects on middle cerebral artery occlusion (MCAO)/reperfusion-induced brain injury via BDNF induction. MCAO animal model is utilized to mimic the condition of ischemic stroke and can be used to assess the function, pathology, and molecular changes in neuronal tissues. Moreover, we also tested the protective effect of LIPUS on ischemia/reperfusion-induced cell injury in cultured microglial cells.

## Results

**Figure 1:**
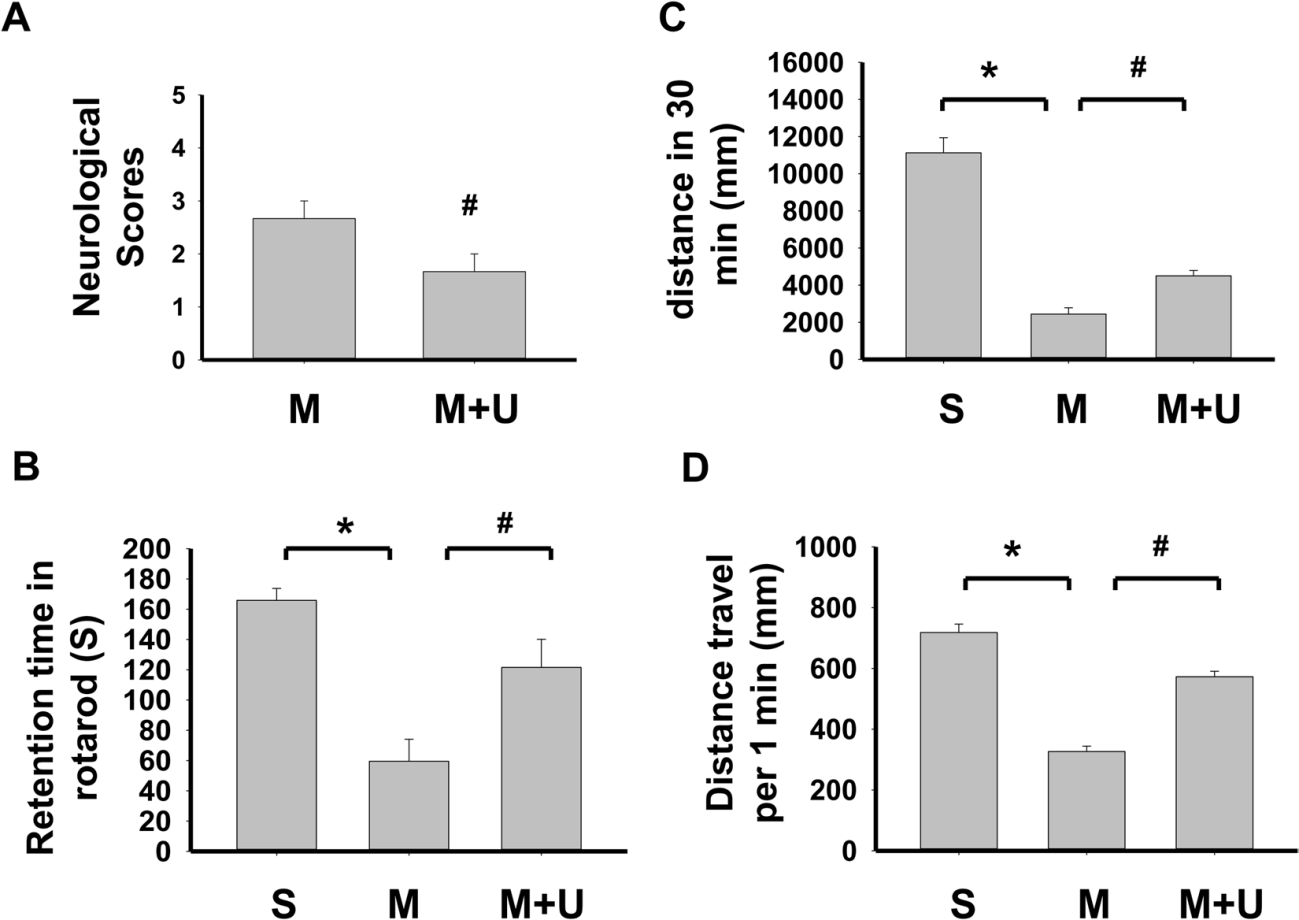
LIPUS pretreatment ameliorated the neuronal functions in mice after MCAO. Mice were pretreated with LIPUS for 5 consecutive days (15 min daily) before MCAO procedure. Neurological function score was assessed 24 h after MCAO (**A**). Animal behavior experiments were tested by rotarod (**B**) or locomotor activity test (**C** and **D**) after MCAO on day 7. Ambulation distance of locomotor activity test was shown in (**C**). The average movement distance was shown in (**D**). Data are presented as means ± SEM (*n* = 6 per group). *P < 0.05, sham group *versus* MCAO group; ^#^*P* < 0.05, MCAO group *versus* MCAO + LIPUS group. S: sham, M: MCAO, M + U: MCAO + LIPUS.

**Figure 2:**
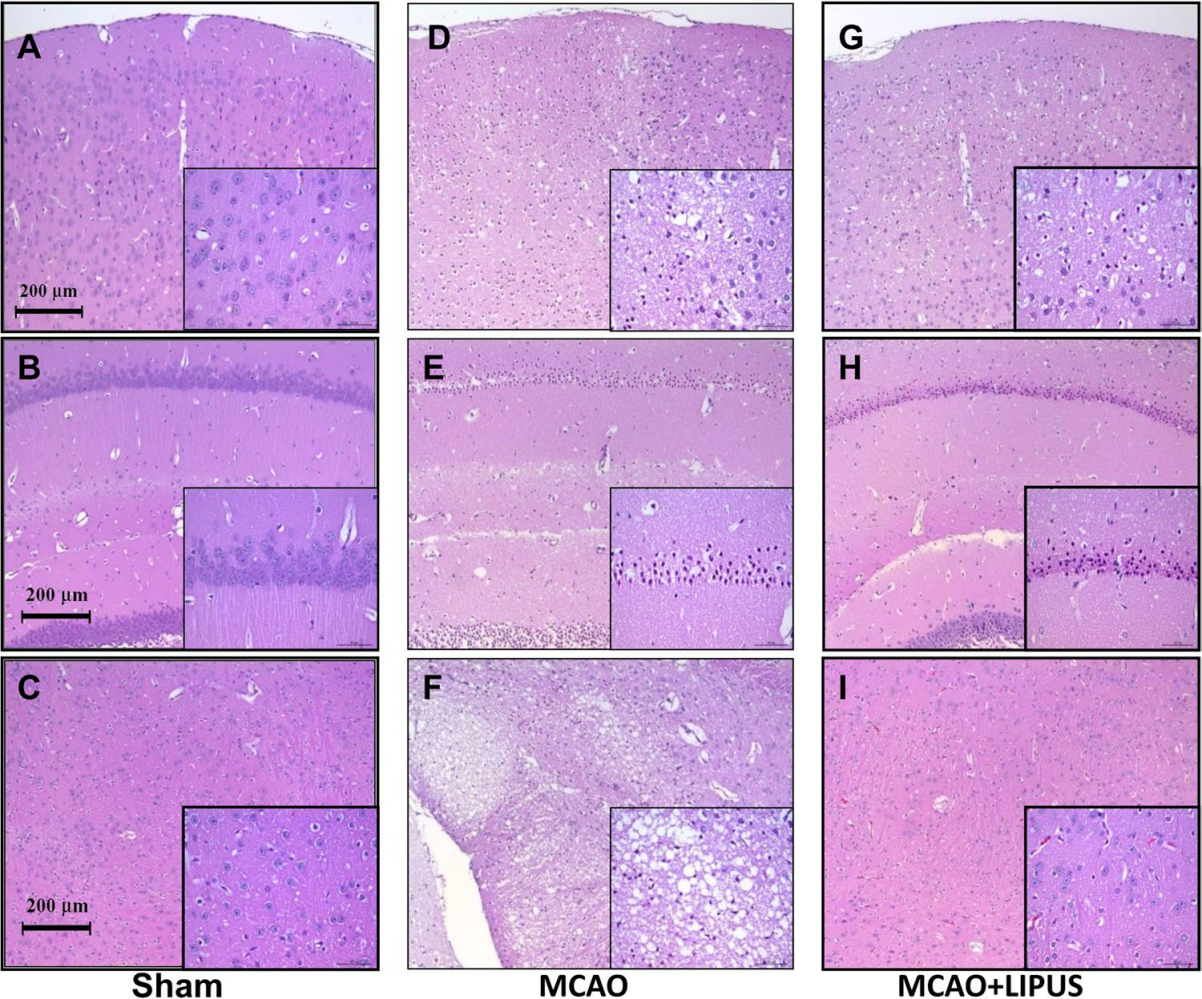
LIPUS pretreatment decreased the pathological changes in brain tissues of mice after MCAO. Mice were pretreatment LIPUS for 5 consecutive days (15 min daily) before MCAO procedure. The pathological changes in left cerebral hemisphere tissues were detected by the H&E staining after MCAO on 7 day. The cerebral cortex, hippocampus, and thalamus of each group were displayed in (**A**,**D**, and **G**), (**B**,**E**, and **H**) and (**C**,**F**, and **I**), respectively. There were six mice per group. The inserted figures in each figure presented the large scale of magnification.

**Figure 3:**
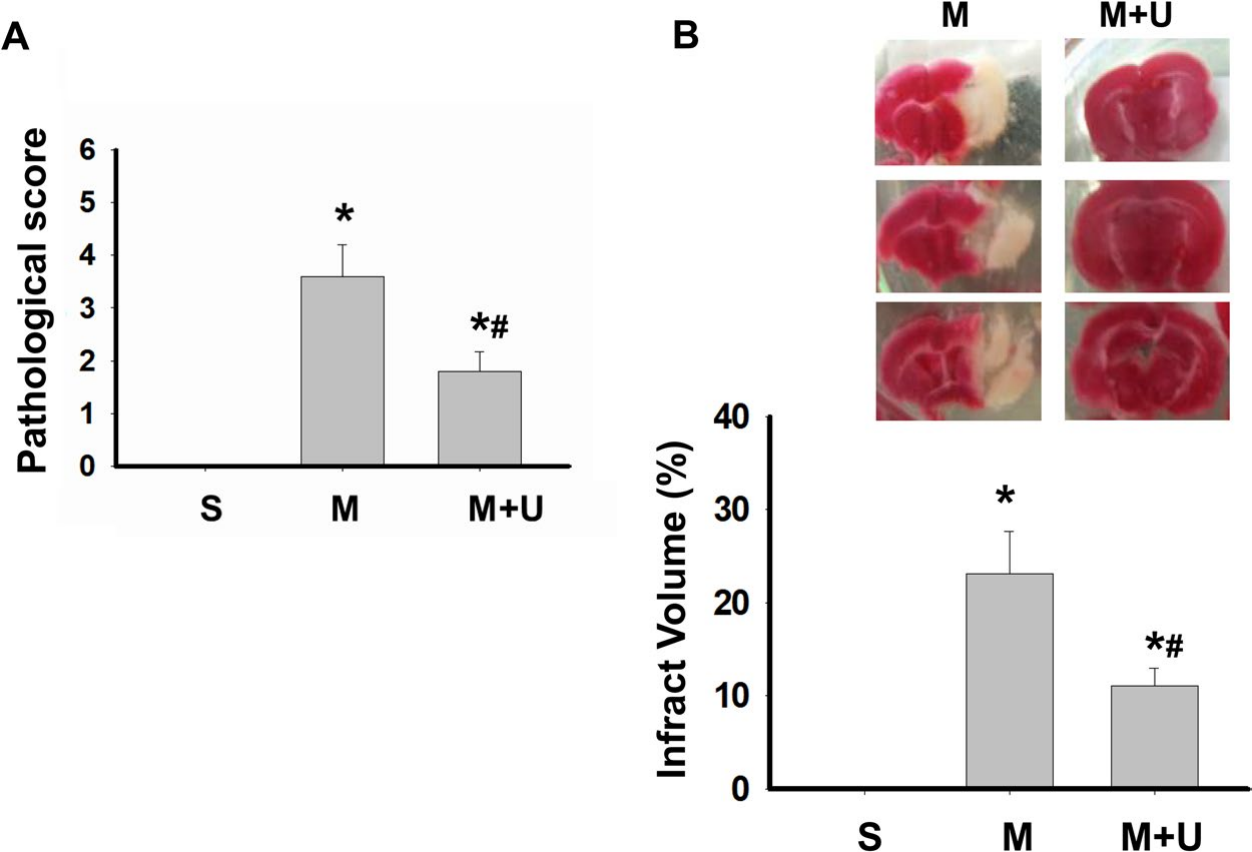
LIPUS pretreatment improved neuronal damage in brain tissue of mice after MCAO. Mice were pretreatment LIPUS for 5 consecutive days (15 min daily) before MCAO procedure. The pathological changes in left cerebral hemisphere tissues were scoring after MCAO on 7 day (**A**). The brain infract volumes were also presented (**B**). Data are presented as means ± SEM (*n* = 6 per group). *P < 0.05, sham group *versus* MCAO group; ^#^*P* < 0.05, MCAO group *versus* MCAO + LIPUS group. S: sham, M: MCAO, M + U: MCAO + LIPUS.

In order to assess the neurological status of mice, we performed locomotor activity test and motor equilibrium performance on rotarod at one week before and after MCAO with or without LIPUS pretreatment. We also evaluated neurological deficit scores after MCAO at 24 h. As shown in Fig. 1A, the neurological deficit scores were significantly decreased in LIPUS-pretreated MCAO mice. Before MCAO, the performances of rotarod and locomotor activity were similar among each group (data not shown). After MCAO administration for one week, the performances of rotarod (Fig. 1B) and locomotor activity (Fig. 1C and D) in mice were impaired, which could be significantly reversed by LIPUS pretreatment (Fig. 1B–D). Moreover, the histopathological examination showed that left cerebral hemisphere in sham group was no histopathological changes in cerebrum (cerebral cortex, hippocampus, thalamus and hypothalamus) (Fig. 2A–C). In left cerebral hemisphere of MCAO group, the cerebrum showed the moderate/severe, unilateral, locally extensive neuronal necrosis and neuronal cell loss with cytoplasmic vacuolation of neuropils (Fig. 2D–F). In MCAO mice pretreated with LIPUS, the hippocampus of cerebrum showed the minimal, unilateral, multifocal neuronal necrosis and vacuolation of neuropils. There was no obviously histopathological change in cerebral cortex, thalamus, and hypothalamus (Fig. 2G–I). The pathological score were also significantly decreased in MCAO mice pretreated with LIPUS (Fig. 3A). Moreover, LIPUS pretreatment significantly counteracted the increased fraction volume in MCAO mice. The percentage of the volume to the contralateral hemisphere was shown in Fig. 3B. These results indicated that pre-administration with LIPUS obviously improved MCAO/reperfusion-induced action ability loss and pathological changes in mice.

**Figure 4:**
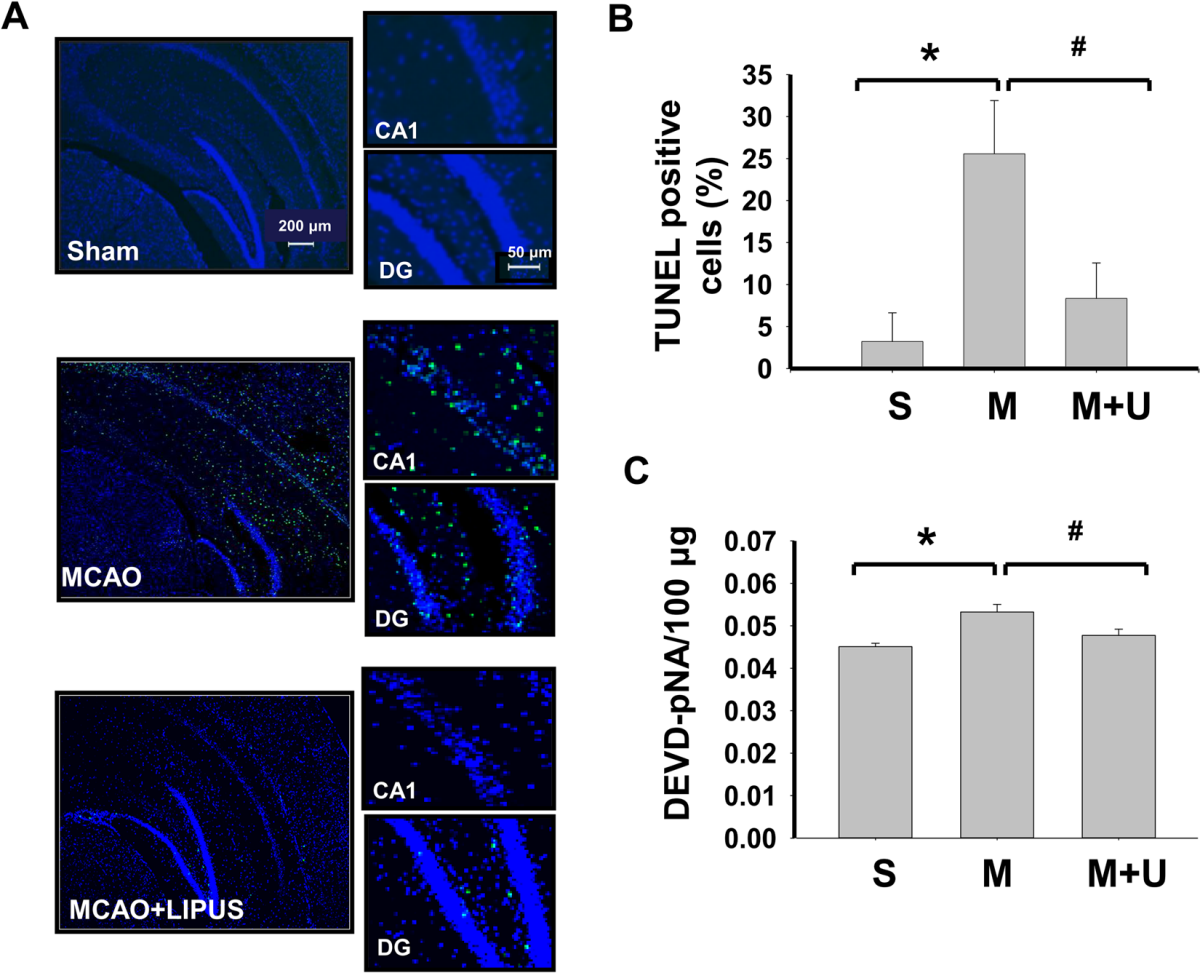
LIPUS pretreatment decreased the cell apoptosis in left cerebral hemisphere tissues of mice after MCAO. Mice were pretreatment LIPUS for 5 consecutive days (15 min daily) before MCAO procedure. The cell apoptosis was performed by the TUNEL staining in the left cerebral hemisphere tissues after MCAO on 7 day. In A, the TUNEL-positive cells were presented as the green color, while cell nuclei were presented as blue color. Scale bar = 200 μm. The magnified images for TUNEL-positive cells in hippocampal CA1 and dentate gyrus (DG) regions were also shown. Scale bar = 50 μm. The TUNEL-positive cells were quantified in (**B**). The caspase-3 activity in brain tissues was also detected (**C**). Data are presented as means ± SEM (*n* = 6 per group). *P < 0.05, sham group *versus* MCAO group; ^#^*P* < 0.05, MCAO group *versus* MCAO + LIPUS group. S: sham, M: MCAO, M + U: MCAO + LIPUS.

**Figure 5:**
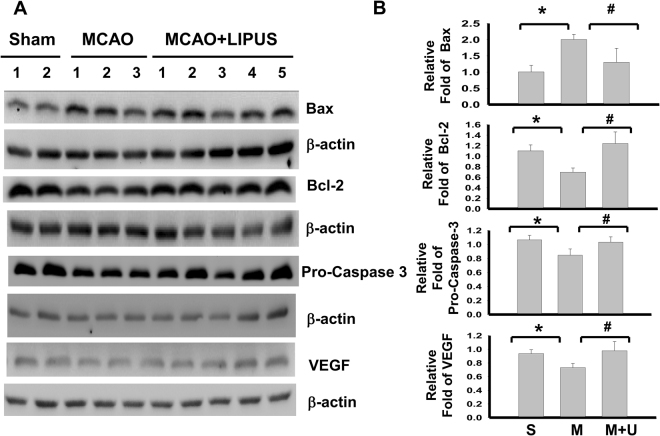
Effects of LIPUS pretreatment on the changes of signaling molecules protein expressions in left cerebral hemisphere tissues of mice after MCAO. Mice were pretreatment LIPUS for 5 consecutive days (15 min daily) before MCAO procedure. The protein expressions of Bax, Bcl-2, pro-caspase-3, and VEGF in the left cerebral hemisphere tissues after MCAO on 7 day were determined by Western blotting (**A**). The densitometric quantifications of proteins expressions were shown in (**B**). Data are presented as means ± SEM (*n* = 3–5 per group). *P < 0.05, sham group *versus* MCAO group; ^#^*P* < 0.05, MCAO group *versus* MCAO + LIPUS group. S: sham, M: MCAO, M + U: MCAO + LIPUS.

**Figure 6:**
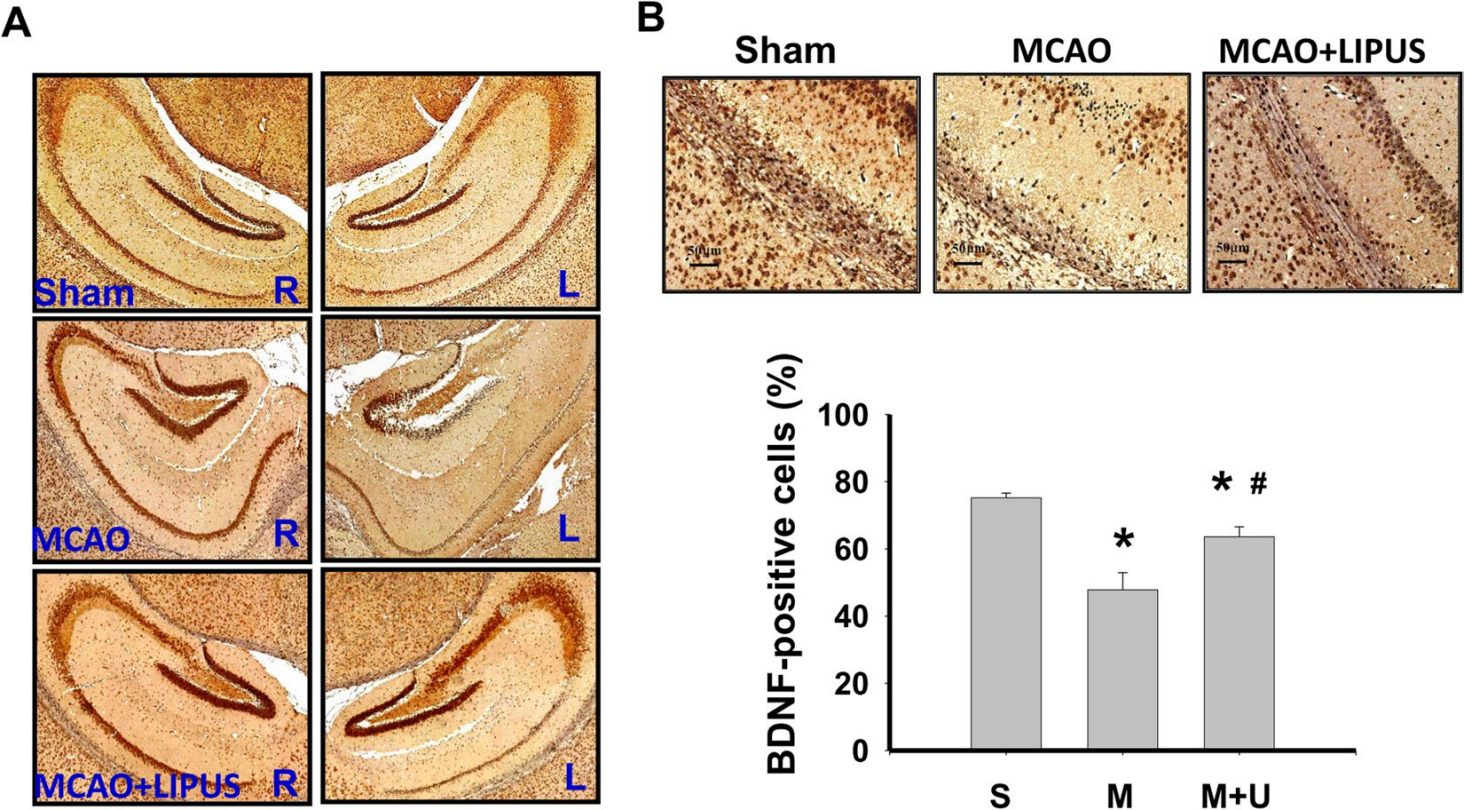
LIPUS pretreatment induced the protein expression of BDNF in left hemispheres of mice after MCAO. Mice were pretreatment LIPUS for 5 consecutive days (15 min daily) before MCAO procedure. The protein expressions of BDNF in both right and left cerebral hemispheres after MCAO on 7 day was determined by immunohistochemical staining (**A**). The BDNF-positive cells in hippocampal CA1 regions were counted (**B**). Data are presented as means ± SEM (*n* = 6 per group). *P < 0.05 sham group *versus* MCAO group; ^#^*P* < 0.05, MCAO group *versus* MCAO + LIPUS group. S: sham, M: MCAO, M + U: MCAO + LIPUS.

Next, we elucidated the effects of LIPUS on the changes of signaling molecules during cerebral injury by MCAO. As shown in Fig. 4, the apoptotic cell death in left cerebral hemisphere of mice was performed by TUNEL staining. There was no obviously TUNEL-positive cells in sham group (Fig. 4A). TUNEL-positive cells were significantly and obviously detected near dentate gyrus and hippocampus in MCAO mice, which could be significantly reversed by LIPUS pretreatment (Fig. 4A and B). Similarly, the caspase-3 activity of brain tissue was also significantly increased in MCAO mice, which could be effectively reversed by LIPUS pretreatment (Fig. 4C). Following, tissues of left cerebral hemisphere were extracted for Western blotting analysis. The increased Bax, decreased Bcl-2, decreased pro-caspase 3, and decreased VEGF protein expressions were shown in brain tissues from MCAO mice. Pretreatment of LIPUS significantly reversed these protein expressions in brain tissues from MCAO mice (Fig. 5A and B). Moreover, the immunohistochemical staining of BDNF showed that BDNF-positive cells were broadly presented in left cerebral hemispheres of sham group, but sparsely scattered BDNF-positive cells were presented in the cerebral cortex and hippocampus of left cerebral hemisphere from MCAO mice, which could be significantly reversed by LIPUS pretreatment (Fig. 6). In the contralateral cerebral hemispheres (right hemisphere), there were normal BDNF protein expressions and no obviously morphological changes in sham, MCAO, and LIPUS + MCAO groups (Fig. 6A). These results indicated that LIPUS pretreatment was capable of preventing the cell apoptotic death and decreased BDNF expression in the brain of MCAO mice.

**Figure 7:**
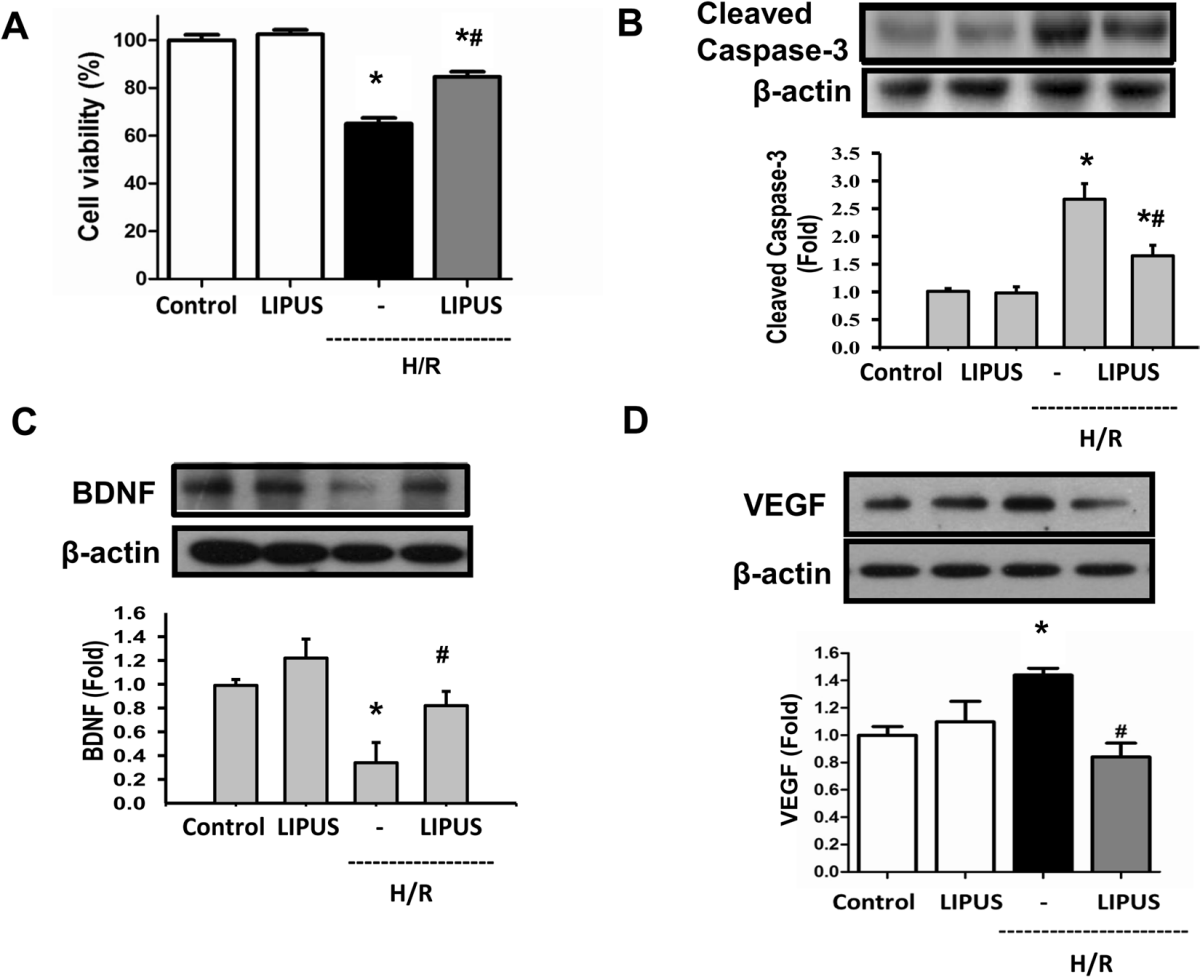
Effects of LIPUS on cell viability and protein expressions of BDNF and VEGF in microglial cells during hypoxia/reperfusion. Microglial BV-2 cells were treated with hypoxia/reperfusion (H/R; hypoxia for 6 h and reperfusion for 12 h) in the presence or absence of LIPUS (110 mW/cm^2^, 15 min before H/R). The cell viability was determined by MTT assay (**A**). The protein expressions of cleaved caspase-3 (**B**) and BDNF (**C**) and VEGF (**D**) were performed by Western blotting. Data are presented as means ± SEM (n = 4). *P < 0.05, control group *versus* H/R group. ^#^*P* < 0.05, H/R group *versus* LIPUS + H/R.

We also evaluated the protective effect of LIPUS on hypoxia/reperfusion-induced cell injury in cultured microglial cells. As shown in Fig. 7A and B, the decreased cell viability and increased caspase-3 cleavage were shown in ischemia/reperfusion-treated BV-2 microglial cells. LIPUS pretreatment significantly reversed the ischemia/reperfusion-induced decreased cell viability and increased caspase-3 cleavage in BV-2 cells (Fig. 7A and B). Moreover, hypoxia/reperfusion downregulated the BDNF protein expression in hypoxia/reperfusion-treated BV-2 microglial cells, which could be significantly reversed by LIPUS pretreatment (Fig. 7C). On the contrary, hypoxia/reperfusion upregulated the VEGF protein expression in hypoxia/reperfusion-treated BV-2 microglial cells, which could be significantly reversed by LIPUS pretreatment (Fig. 7D). These results indicated that LIPUS was capable of inducing BDNF expression and antagonizing hypoxia/reperfusion-induced microglial cell injury.

## Discussion

In the present study, we demonstrated the protective effect of LIPUS against experimental cerebral ischemia/reperfusion injury. The condition of LIPUS in this study is same as our previous reports^12,18^, although there is a different time course. The LIPUS condition is 0.51 W acoustic power, I_SPTA_ 528 mW/cm^2^, for 5 days (this study), 42–49 days^12^, or 3–27 days^18^. In the studies of Yang *et al*.^12^ and Su *et al*.^18^, they observed that there were no obviously neural apoptotic death, inflammatory cell infiltration, and behavioral changes in rodent animals treated with LIPUS alone. Therefore, there is a safe condition for LIPUS exposure in this study. On the other hand, our previous study has found that after LIPUS treatment for 3 consecutive days, BDNF and VEGF can be activated in a mouse traumatic brain injury model^19^. Hence, we determined the duration of LIPUS treatment in MCAO model is longer than 3 days at least. Our preliminary experiments showed that pretreatment of LIPUS for 5 consecutive days could get a better protective effect on MCAO model. Moreover, the surgical injury of MCAO leads the weakness or even death in mice during 72 h that is hard to determine the improvement of neurological and behavioral changes by LIPUS. Our preliminary experiments showed that the neurological and behavioral alterations and the molecular biological changes at 7 day after MCAO could be a proper time course to determine the LIPUS-induced protective effects against MCAO-induced brain injury.

So far, stroke still performs highly mortality, morbidity, and recurrence in worldwide countries. Reperfusion therapies like as thrombolysis using recombinant tissue plasminogen activator are currently effective treatment for ischemic stroke^20^. Yet, the healing patients remain less than 5–10% of the population because of the therapeutic window restriction for 6 h^21^. Furthermore, the development of pharmacological neuroprotective treatment is disappointing when translating from experimental studies to clinical studies. Therapeutic ultrasound has been applied to therapy the ischemic stroke combined with the enzymatic mediated thrombolysis^1,12,14^. A previous experimental study has indicated that transcranial pulsed ultrasound administration was capable of stimulating intact brain circuits^9^. Recently, Guo *et al*. have found that rats subjected to 60 min of LIPUS stimulation (with spatial average pulse average intensity of 2.155 W/cm^2^ and spatial average temporal average of 86 mW/cm^2^) immediately after the ischemia (MCAO) can protect brain from stroke injury^22^.

Nevertheless, prevention is better than cure. Li and colleagues (2017) have recently indicated that LIPUS preconditioning 60 min with 400 mW/cm^2^ and 500 kHz moderated focal cerebral ischemia in rats; they found that histopathology outcomes, including the infract volumes (ischemic area) and ipsilateral hemisphere volumes, were effectively improved at 24 and 48 h after brain ischemia reperfusion^23^. However, Li and colleagues (2017) did not further clarify the type of brain cell injury (eg. apoptosis) and the possible mechanism by LIPUS. In the present study, we utilized a short-term but consecutive pre-ischemic treatment with LIPUS (15 min daily for 5 days, 528 mW/cm^2^ before MCAO procedure) to MCAO mice. We found that the pretreatment of LIPUS significantly ameliorated the ischemic brain injury, as evidence of reducing neurologic deficit scores, less infarct area, decreasing pathological score, and better neurological functions and behavior performance (the motor equilibrium performance on rotarod and locomotor activity) in MCAO mice. We further demonstrated that pre-ischemic treatment with LIPUS mitigated the apoptotic cell death and increased the BDNF expression in brain tissues and cultured microglial cells. Together with these results indicate that LIPUS pretreatment possesses the improvement of neuron injury and the moderation of disease condition of ischemic stroke through the inhibition of cell apoptosis and the induction of neuronal protective factor BDNF.

Inflammation induction is a key symptom in the stroke process. Growing evidence suggested that down-regulation of inflammation after stroke could decrease the ischemic injuries and improve outcomes^24^. Guo *et al*. have also found that LIPUS induces the neuroprotective effect on brain ischemia might be due to the increased cerebral blood flow and reduction of neutrophils^22^. In the present study, the ischemic brain showed moderate/severe, unilateral, locally extensive neuronal necrosis, and neuronal cell loss with vacuolation of neuropils in histopathological sections. Pretreatment with LIPUS effectively reduced the brain damage to minimal, unilateral, multifocal neuronal necrosis and vacuolation of neuropil in hippocampus, and no significant histopathological changes of cerebral cortex, thalamus and hypothalamus (Fig. 2). From the findings of Guo’s *et al*. and the present work, both therapeutic utilization (after stroke) and preventive treatment (before stroke) of LIPUS in the MCAO mice reduced neutrophils number and moderated the inflammatory responses after brain ischemia.

Several important neuronal factors are known to protect against ischemic injury in MCAO mice and considered as potential therapeutic target such as VEGF and BDNF. VEGF is associated with angiogenesis and increase of vascular permeability, neurogenesis, and neuronal survival^25^. Evidence indicated the increase of VEGF in the early stage (day1 to day3) of MCAO promotes angiogenesis and neuronal function recovery^26,27^. Unexpectedly, in the present study, we found that VEGF was significantly decreased at day 7 after ischemic stroke surgical treatment, which was reversed by LIPUS administration. We guessed that the decrease of VEGF may result from the tissue damages like as endothelial cell injury-diminished VEGF expression in the late stage after MCAO treatment. We will address this possibility in the further study. On the other hand, it has been suggested that microglial cells are one of the major cell types to express VEGF in an MCAO animal model^28^. Recently, Kanazawa *et al*. showed that the level of secretory VEGF from conditioned media of microglia subjected to oxygen-glucose deprivation was markedly increased^29^. Similarly, in this study, we also found that hypoxia/reperfusion upregulated the VEGF protein expression in hypoxia/reperfusion-treated microglial cells. The increased VEGF expression could be significantly reversed by LIPUS pretreatment. These results indicate that LIPUS may be capable of improving the functions of microglia under hypoxia/reperfusion condition or ischemic brain injury.

Another neurotrophic protective factor, BDNF, is known to be involved in synaptic plasticity^30,31^ and neural circuit function^32,33^. Huang *et al*. have found that LIPUS stimulation protects against brain injury in the hippocampus and corpus callosum in experimental vascular dementia rats, which the beneficial effect of LIPUS may be partly attributed to the up-regulation of BDNF production^13^. Several proposed actions and mechanisms of BDNF were suggested for its beneficial effects on stroke recovery, including protecting against acute ischemic injury^34,35^, inducing angiogenesis^36^, enhancing neurogenesis^37^, increasing brain repair^38^, and promoting synaptic plasticity^31,39^, Moreover, some studies also linked the enhancing BDNF production to the post-stroke recovery^3,40,41^. In the present study, we observed that pretreatment with LIPUS significantly attenuated MCAO/reperfusion-induced decrease of BDNF in brain tissues of mice. This finding supported the neuroprotective effect of LIPUS pretreatment on MCAO mice via the prevention of decrease in the BDNF production.

LIPUS has been shown to enhance cell proliferation and neurotrophin-3 gene expression in Schwann cells isolated from rat sciatic nerve^42^. Yang *et al*. have shown that LIPUS stimulation can increase the protein expressions of BDNF, GDNF, and VEGF in cultured rat brain astrocytes, and further demonstrated that LIPUS enhanced the neurotrophic factors and protected against chemical (AlCl_3_)-induced cerebral damages as well as astrocyte apoptotic death in rats^12^. It has been found that LIPUS stimulation could enhance BDNF expression in astrocytes via the molecular mechanisms involved TrkB/PI3K/Akt and calcium/CaMK signaling pathways^43^. Moreover, the non-neuronal cells have been shown to be capable of producing BDNF in the brain after ischemic stroke in rats^17^. The white matter-activated glial cells have been suggested to play an important role in protecting nerve fibers in the ischemic monkey brain by producing BDNF^44^. The activated microglial cells were able to trigger the BDNF production under an inflammatory condition^45^. In this study, we also found that LIPUS stimulation significantly inhibited the ischemia/reperfusion-induced cell apoptosis and cell injury and was capable of increasing the BDNF protein expression in cultured microglial cells. Taken together, the previous findings^12,43^ and the present results indicate that LIPUS can stimulate the BDNF production in non-neuronal cells (astrocyte and microglia). The BDNF production from non-neuronal cells may be involved in protecting ischemic brain injury in MCAO animal model. However, the role of BDNF producing from non-neuronal cells in protecting against ischemic brain injury by LIPUS still remains unclear because there is a lack of *in vivo* evidence. The cellular source of the observed changes in MCAO animal model in the presence or absence of LIPUS exposure needs further investigation in the future.

In conclusion, this study demonstrated for the first time that pretreatment with LIPUS by 15 min daily for 5 days effectively ameliorated the brain damage via BDNF induction in an ischemic stroke mouse model. Molecular evidence showed that LIPUS pretreatment improved apoptotic cell death and increased BDNF production in peri-ischemic brain tissues of MCAO mice. These findings suggest that LIPUS treatment may be potentially applied to prevent ischemic stroke or other neuronal diseases and serves as a novel prevention strategy in the clinical.

## Materials and Methods

### Animals

Six-week-old male C57BL/6 mice were provided by Laboratory Animal Center, College of Medicine, National Taiwan University (Taipei, Taiwan). The Institutional Animal Care and Use Committee of the College of Medicine, National Taiwan University, approved and conducted the animal study in accordance with the regulations of Taiwan and NIH guidelines on the care and welfare of laboratory animals. The animals were treated humanely and with regard for alleviation of suffering. Mice were housed in a room at a constant temperature of 21 ± 2 °C and a 12 h light/dark cycle until experimental use.

### Middle cerebral artery occlusion (MCAO) procedure

Transient focal cerebral ischemia was induced by MCAO using an intraluminal filament technique as described in our previous study^46^. Briefly, C57BL/6 mice were anaesthetized by intraperitoneal injection of ketamine (100 mg/kg) and xylazine (10 mg/kg) (Sigma-Aldrich, Louis, MO, USA) and maintained the rectal temperature at 37 ± 0.5 °C. Firstly, after the dissection of neck and carotid bifurcation, identified the left common carotid artery and used the external carotid artery as a stump. A thread with 6–0-nylon was used to insert through the incision in the external carotid artery stump to the origin of the middle cerebral artery. The filament tip coated with ethyl cyanoacrylate polymer was left in the position for 45 min to occlude the blood supply. A Laser Doppler (PeriFlux 4001, Perimed, Stockholm, Sweden) was used to monitor the cerebral blood flow during MCAO. The cerebral blood flow was reduced to less than 30% of the pre-ischemic value. At the end of the procedure, the filament was withdrawn and the incision was sutured. Animals were cared and monitored until full recovery from anesthesia.

### LIPUS treatment

The pulsed ultrasound setup was performed as described previously^12,18^. A 1.0-MHz single-element focused transducer (A392S, Panametrics, Waltham, MA, USA) with a diameter of 38 mm and a radius of curvature of 63.5 mm was used to generate the LIPUS exposure and was applied with a 5% duty cycle and a repetition frequency of 1 Hz. This transducer was mounted on a removable cone filled with deionized and degassed water. The cone tip was capped by a polyurethane membrane and the focal zone center was about 2.0 mm away from the tip. The transducer was positioned by using a stereotaxic apparatus to direct the acoustic beam to the target region in the brain of mice under anesthesia with isoflurane mixed with oxygen. An acoustic power of 0.51 W (corresponding to a spatial-peak temporal-average intensity of 528 mW/cm^2^) was applied to the target region in the injured cortical areas with 15 min daily (a 5-minute interval between each sonication) for 5 days before MCAO treatment. The LIPUS exposures duration was based on the data of previous study^19^.

### Neurological deficits evaluation

Neurological deficits were evaluated at 24 h after MCAO procedure as follows with a 4-point scale adapted and modified from Bederson’s neurological scoring scale:^47^ 0, no deficit; 1, forelimb weakness and turning to the ipsilateral side when held by tail; 2, circling to the contralateral side; 3, unable to bear weight on affected side; and 4, no spontaneous motor activity.

### Locomotor activity test

The locomotor activity boxes (9 × 20 × 11 cm) (Imetronic, Bordeaux, France) were used to test the locomotor activity responses. There are two lines of photocells in the boxes in which one is 2 cm above the floor to measure horizontal activity and the other is 6 cm above the floor to measure vertical activity (rears) with a low luminosity environment (5 lux). Mice were habituated to the locomotor activity boxes for 30 min before experimental procedure. The locomotor activity test for all mice was carried out one week before and after getting the MCAO procedure.

### Motor equilibrium performance on rotarod

The rotarod test was used to test the motor equilibrium performance in mice with a slowly rotating rod (60 revolutions per min). Mice were tested through ten consecutive sessions to stay on the rod and reach the cut off time of 180 s one week before getting the MCAO procedure. Mice were tested again one week after getting the MCAO. The retention time, which defined as total time (sec) remaining on the rod, was recorded at each session.

### Histopathological and immunohistochemical analysis

The paraffin-embedded brain tissues were horizontally sliced to make the paraffin sections that were analyzed with hematoxylin and eosin (H&E) staining or immunohistochemical staining. Severity of lesions was graded as previously described by Shackelford *et al*.^48^. The 4-μm sections of cerebral samples were stained with H&E reagent. Degree of lesions was graded from one to five depending on severity: 1 = minimal (<1%); 2: slight (1–25%); 3 = moderate (26–50%); 4 = moderate/severe (51–75%); 5 = severe/high (76–100%). Moreover, immunohistochemical staining was performed as previously described^49^. The DAB detection kit (Biogenex, Fremont, CA, USA) and primary rabbit polyclonal anti-BDNF antibody (Santa Cruz, Santa Cruz, CA, USA) was used. The staining was observed under an upright microscope and manually counted for 5 random fields. BDNF positive cells, expressing in hippocampal CA1 region, were counted by using Fovea Pro 4.0 software.

### Measurement of infarct volume

Mice were sacrificed under anesthesia after MCAO procedure. Coronal slices of brain were cut at 1–2 mm from the frontal tips. The sections were immersed in 2% 2,3,5-triphenyltetrazolium chloride at 37 °C for 20 min. Upon completion of the staining procedure, the sections were examined for areas, which did not take up the staining. The non-stained areas were considered as infarcted. Infarction areas were measured on the rostral and caudal surfaces of each slice and numerically integrated across the thickness of the slice to obtain an estimate of infarct volume. The volumes were summed to calculate the infarct volume over the entire hemisphere, which expressed as the percentage of the volume of the contralateral hemisphere. This procedure was analyzed by SigmaScan Pro 5.0 software (SPSS, Chicago, IL, USA).To correct the swelling, the volumes of the ipsilateral and contralateral hemispheres were compared.

### Western blotting

Mice (Sham, MCAO, and MCAO/LIPUS groups) were sacrificed and the left cerebral hemispheres (the damaged hemisphere for MCAO) of brain tissues were isolated. Tissues were homogenized in RIPA buffer at 4 °C, centrifuged at 12000 × g for 30 min. Equal amounts of proteins from supernatant were subjected to SDS-PAGE and blotted onto polyvinylidene difluoride (PVDF; Millipore, Billerica, MA, USA). The PVDF films were first blocking with 5% skimmed milk/PBS (50 mM Tris-HCl, pH 7.5, 150 mM NaCl, 0.1% Tween 20) buffer for 1 h, and then incubated overnight at 4 °C with primary antibodies for Bcl-2, Bax, BDNF, VEGF (Santa Cruz), caspase-3 (Abcam, Cambridge, MA, USA), and β-actin (Sigma-Aldrich). After washing, the films were incubated with horseradish peroxidase-conjugated secondary antibodies, followed by detection using enhanced chemiluminescence. Densitometric quantification was performed by using GelDoc Image software (Scion, Frederick, MD, USA) to quantify protein expression.

### Terminal deoxynucleotidyl transferase (TdT) dUTP nick end labeling (TUNEL) assay

The left cerebral hemispheres of brain tissues were isolated for TUNEL assay. The frozen tissue sections (5 μm) were dried and immersed in 1% sodium hydroxide 80% ethanol buffer for 5 min, rinsed in 70% ethanol for 2 min, and then rinsed in distilled water. After phosphate-buffer saline (PBS) washing for three times, sections were staining with the Terminal deoxynucleotidyl transferase-mediated biotinylated UTP nick end labeling (TUNEL; Promega, Madison, WI, USA) and the staining procedure was followed to the manufacturer’s protocol. After TUNEL staining, the sections were rinsed in PBS, incubated with ammonium chloride for 20 min and detected by Hoechst 33258 (1 mg/ml; Sigma-Aldrich) counter stain. The number of TUNEL-positive cell was counted in 10 randomly selected visual fields under 200x magnification.

### Caspase-3 activity assay

The left cerebral hemispheres of brain tissues were isolated for caspase-3 activity assay. The tissues were homogenized, and then centrifuged at 20,000 × g for 15 min at 4 °C. Activity of caspase-3 was measured using a caspase-3 activity test kit (Abcam). According to the manufacturer’s instructions, 100 μg of total proteins in 100 μl total volumes was mixed with 100 μl of equilibrated test Caspase-Glo reagent and incubated for 1 h at 37 °C. The caspase-3 enzyme activity was measured using a luminometer reader (Beckman Co., Brea, CA, USA).

### Cell culture and hypoxia/reperfusion (H/R) treatment

Microglial cell line BV2 cells were cultured as described previously^50^. Briefly, BV2 cells were cultured in the Dulbecco’s Modified Eagle’s Medium (DMEM; GIBCO, Grand Island, NY, USA), containing with 10% fetal bovine serum (FBS; GIBCO) and 1% antibiotics (100 IU/mL penicillin, 100 µg/ml streptomycin, 0.0025 mg/ml Amphotericin B; GIBCO) at 37 °C under 5% CO_2_. They were transferred to fresh complete medium when growing to 80% cell confluence. To mimic H/R-induced injury, BV2 cells were pre-treatment with LIPUS for 15 min (intensity: 110 mW/cm^2^) transferred to the deprivation medium, put cells in the hypoxia tank which contained anaerobic bags for 6 h and then recovered in the complete medium for 12 h. Total proteins were collected and used to the following experiments.

### Cell viability assay

BV2 cells were cultured in the 96-well plates for 24 h. BV2 cells were cultured in the 3-(4,5-dimethylthiazol-2-yl)-2,5-diphenyltetrazolium bromide (MTT)-contained medium at 37 °C for 2 h. The formazan crystals were formed, which were completely dissolved by adding DMSO. The absorbance was detected at 540 nm.

### Statistics

Data are expressed as mean ± standard error of the means of at least three independent experiments. The statistical significance of differences in the means of experimental groups were analyzed by one-way analysis of variance (ANOVA) and unpaired two-tailed Student’s *t*-test with a significance threshold of 0.05 via GraphPad Prism software.

## References

[1] Mozaffarian D (2016). Heart Disease and Stroke Statistics-2016 Update: A Report From the American Heart Association. Circulation.

[2] World Health Organization The top 10 causes of death Updated January http://www.hoint/mediacentre/factsheets/fs310/en/ Accessed September 1, 2017, (WHO report) (2017).

[3] Berretta A, Tzeng YC, Clarkson AN (2014). Post-stroke recovery: the role of activity-dependent release of brain-derived neurotrophic factor. Expert Rev Neurother.

[4] Wardlaw, J. M., Zoppo, G., Yamaguchi, T. & Berge, E. Thrombolysis for acute ischaemic stroke. *Cochrane Database Syst Rev*, CD000213 (2003). 10.1002/14651858.CD000213 12917889

[5] Piper RJ, Hughes MA, Moran CM, Kandasamy J (2016). Focused ultrasound as a non-invasive intervention for neurological disease: a review. Br J Neurosurg.

[6] Xin Z (2016). Clinical applications of low-intensity pulsed ultrasound and its potential role in urology. Transl Androl Urol.

[7] Lu H (2006). Low-intensity pulsed ultrasound accelerates bone-tendon junction healing: a partial patellectomy model in rabbits. Am J Sports Med.

[8] Pomini KT (2014). Effect of low-intensity pulsed ultrasound on bone regeneration: biochemical and radiologic analyses. J Ultrasound Med.

[9] Crisci AR, Ferreira AL (2002). Low-intensity pulsed ultrasound accelerates the regeneration of the sciatic nerve after neurotomy in rats. Ultrasound Med Biol.

[10] Chang CJ (2005). Low-intensity-ultrasound-accelerated nerve regeneration using cell-seeded poly(D,L-lactic acid-co-glycolic acid) conduits: an *in vivo* and *in vitro* study. J Biomed Mater Res B Appl Biomater.

[11] Tufail Y (2010). Transcranial pulsed ultrasound stimulates intact brain circuits. Neuron.

[12] Yang FY (2015). Enhancement of Neurotrophic Factors in Astrocyte for Neuroprotective Effects in Brain Disorders Using Low-intensity Pulsed Ultrasound Stimulation. Brain Stimul.

[13] Huang SL, Chang CW, Lee YH, Yang FY (2017). Protective Effect of Low-Intensity Pulsed Ultrasound on Memory Impairment and Brain Damage in a Rat Model of Vascular Dementia. Radiology.

[14] Daffertshofer M, Hennerici M (2003). Ultrasound in the treatment of ischaemic stroke. Lancet Neurol.

[15] Daffertshofer M, Fatar M (2002). Therapeutic ultrasound in ischemic stroke treatment: experimental evidence. Eur J Ultrasound.

[16] Tsivgoulis G, Culp WC, Alexandrov AV (2008). Ultrasound enhanced thrombolysis in acute arterial ischemia. Ultrasonics.

[17] Bejot Y (2011). Time-dependent contribution of non neuronal cells to BDNF production after ischemic stroke in rats. Neurochem Int.

[18] Su WS, Wu CH, Chen SF, Yang FY (2017). Low-intensity pulsed ultrasound improves behavioral and histological outcomes after experimental traumatic brain injury. Sci Rep.

[19] Su WS, Wu CH, Chen SF, Yang FY (2017). Transcranial ultrasound stimulation promotes brain-derived neurotrophic factor and reduces apoptosis in a mouse model of traumatic brain injury. Brain Stimul.

[20] Hacke W (2008). Thrombolysis with alteplase 3 to 4.5 hours after acute ischemic stroke. N Engl J Med.

[21] Wahlgren N (2016). Mechanical thrombectomy in acute ischemic stroke: Consensus statement by ESO-Karolinska Stroke Update 2014/2015, supported by ESO, ESMINT, ESNR and EAN. Int J Stroke.

[22] Guo T (2015). Pulsed Transcranial Ultrasound Stimulation Immediately After The Ischemic Brain Injury is Neuroprotective. IEEE Trans Biomed Eng.

[23] Li H (2017). Low-intensity (400 mW/cm(2), 500 kHz) pulsed transcranial ultrasound preconditioning may mitigate focal cerebral ischemia in rats. Brain Stimul.

[24] Chen J (2016). Anti-Inflammation of Natural Components from Medicinal Plants at Low Concentrations in Brain via Inhibiting Neutrophil Infiltration after Stroke. Mediators Inflamm.

[25] Greenberg DA, Jin K (2013). Vascular endothelial growth factors (VEGFs) and stroke. Cell Mol Life Sci.

[26] Yang JP, Liu HJ, Liu XF (2010). VEGF promotes angiogenesis and functional recovery in stroke rats. J Invest Surg.

[27] Harms KM, Li L, Cunningham LA (2010). Murine neural stem/progenitor cells protect neurons against ischemia by HIF-1alpha-regulated VEGF signaling. PLoS One.

[28] Plate KH (1999). Cell type specific upregulation of vascular endothelial growth factor in an MCA-occlusion model of cerebral infarct. J Neuropathol Exp Neurol.

[29] Kanazawa M (2017). Microglia preconditioned by oxygen-glucose deprivation promote functional recovery in ischemic rats. Sci Rep.

[30] Greenberg ME, Xu B, Lu B, Hempstead BL (2009). New insights in the biology of BDNF synthesis and release: implications in CNS function. J Neurosci.

[31] Clarkson AN (2011). AMPA receptor-induced local brain-derived neurotrophic factor signaling mediates motor recovery after stroke. J Neurosci.

[32] Minichiello L (2009). TrkB signalling pathways in LTP and learning. Nat Rev Neurosci.

[33] Monteggia LM (2004). Essential role of brain-derived neurotrophic factor in adult hippocampal function. Proc Natl Acad Sci USA.

[34] Ferrer I (2001). Brain-derived neurotrophic factor reduces cortical cell death by ischemia after middle cerebral artery occlusion in the rat. Acta Neuropathol.

[35] Schabitz WR, Schwab S, Spranger M, Hacke W (1997). Intraventricular brain-derived neurotrophic factor reduces infarct size after focal cerebral ischemia in rats. J Cereb Blood Flow Metab.

[36] Kermani P, Hempstead B (2007). Brain-derived neurotrophic factor: a newly described mediator of angiogenesis. Trends Cardiovasc Med.

[37] Schabitz WR (2007). Intravenous brain-derived neurotrophic factor enhances poststroke sensorimotor recovery and stimulates neurogenesis. Stroke.

[38] Mamounas LA (2000). BDNF promotes the regenerative sprouting, but not survival, of injured serotonergic axons in the adult rat brain. J Neurosci.

[39] Waterhouse EG, Xu B (2009). New insights into the role of brain-derived neurotrophic factor in synaptic plasticity. Mol Cell Neurosci.

[40] Vaynman S, Ying Z, Gomez-Pinilla F (2004). Hippocampal BDNF mediates the efficacy of exercise on synaptic plasticity and cognition. Eur J Neurosci.

[41] Knaepen K, Goekint M, Heyman EM, Meeusen R (2010). Neuroplasticity - exercise-induced response of peripheral brain-derived neurotrophic factor: a systematic review of experimental studies in human subjects. Sports Med.

[42] Zhang H, Lin X, Wan H, Li JH, Li JM (2009). Effect of low-intensity pulsed ultrasound on the expression of neurotrophin-3 and brain-derived neurotrophic factor in cultured Schwann cells. Microsurgery.

[43] Liu SH, Lai YL, Chen BL, Yang FY (2017). Ultrasound Enhances the Expression of Brain-Derived Neurotrophic Factor in Astrocyte Through Activation of TrkB-Akt and Calcium-CaMK Signaling Pathways. Cereb Cortex.

[44] Sato Y (2009). White matter activated glial cells produce BDNF in a stroke model of monkeys. Neurosci Res.

[45] Gomes C (2013). Activation of microglial cells triggers a release of brain-derived neurotrophic factor (BDNF) inducing their proliferation in an adenosine A2A receptor-dependent manner: A2A receptor blockade prevents BDNF release and proliferation of microglia. J Neuroinflammation.

[46] Chen CM, Liu SH, Lin-Shiau SY (2007). Honokiol, a neuroprotectant against mouse cerebral ischaemia, mediated by preserving Na+, K+-ATPase activity and mitochondrial functions. Basic Clin Pharmacol Toxicol.

[47] Bederson JB (1986). Rat middle cerebral artery occlusion: evaluation of the model and development of a neurologic examination. Stroke.

[48] Shackelford C (2002). Qualitative and quantitative analysis of nonneoplastic lesions in toxicology studies. Toxicol Pathol.

[49] Xiong JY (2015). Long-term treadmill exercise improves spatial memory of male APPswe/PS1dE9 mice by regulation of BDNF expression and microglia activation. Biol Sport.

[50] Chen CM (2016). Green Tea Catechin Prevents Hypoxia/Reperfusion-Evoked Oxidative Stress-Regulated Autophagy-Activated Apoptosis and Cell Death in Microglial Cells. J Agric Food Chem.

